# Neuropilin-1 Promotes Epithelial-to-Mesenchymal Transition by Stimulating Nuclear Factor-Kappa B and Is Associated with Poor Prognosis in Human Oral Squamous Cell Carcinoma

**DOI:** 10.1371/journal.pone.0101931

**Published:** 2014-07-07

**Authors:** Weiming Chu, Xiaomeng Song, Xueming Yang, Lu Ma, Jiang Zhu, Mengying He, Zilu Wang, Yunong Wu

**Affiliations:** 1 Institute of Stomatology, Nanjing Medical University, Nanjing, PR China; 2 Department of Oral and Maxillofacial Surgery, Stomatological Hospital of Jiangsu Province, Nanjing, PR China; 3 Department of Basic Science of Stomatology, College of Stomatology, Nanjing Medical University, Nanjing, PR China; Seoul National University, Republic of Korea

## Abstract

**Background:**

The epithelial-to-mesenchymal transition (EMT) is a key process in carcinogenesis, invasion, and metastasis of oral squamous cell carcinoma (OSCC). In our previous studies, we found that neuropilin-1 (NRP1) is overexpressed in tongue squamous cell carcinoma and that this overexpression is associated with cell migration and invasion. Nuclear factor-kappa B (NF-κB) plays an essential role both in the induction and the maintenance of EMT and tumor metastasis. Therefore, we hypothesized that NRP1 induces EMT, and that NRP1-induced migration and invasion may be an important mechanism for promoting invasion and metastasis of OSCC through NF-κB activation.

**Methods/Results:**

The variations in gene and protein expression and the changes in the biological behavior of OSCC cell lines transfected with a vector encoding NRP1, or the corresponding vector control, were evaluated. NRP1 overexpression promoted EMT and was associated with enhanced invasive and metastatic properties. Furthermore, the induction of EMT promoted the acquisition of some cancer stem cell (CSC)-like characteristics in OSCC cells. We addressed whether selective inhibition of NF-κB suppresses the NRP1-mediated EMT by treating cells with pyrrolidinedithiocarbamate ammonium (PDTC), an inhibitor of NF-κB. Immunohistochemical analysis of NRP1 in OSCC tissue samples further supported a key mediator role for NRP1 in tumor progression, lymph node metastasis, and indicated that NRP1 is a predictor for poor prognosis in OSCC patients.

**Conclusion:**

Our results indicate that NRP1 may regulate the EMT process in OSCC cell lines through NF-κB activation, and that higher NRP1 expression levels are associated with lymph node metastasis and poor prognosis in OSCC patients. Further investigation of the role of NRP1 in tumorigenesis may help identify novel targets for the prevention and therapy of oral cancers.

## Introduction

Oral squamous cell carcinoma (OSCC) is a major cause of morbidity and mortality worldwide, accounting for at least 90% of all oral malignancies. OSCC is a significant public health concern worldwide as the 5-year relative survival rate has remained lower than 60% over the past four decades [1]. The mechanisms that drive -tumor metastasis, invasion, and drug resistance are critical survival-influencing factors in OSCC, are still complex and currently, poorly understood. Accumulating lines of evidence have shown that the epithelial-to-mesenchymal transition (EMT) contributes to tumor metastasis and invasion [2]–[5]. EMT is mediated by the down-regulation of epithelial cell-specific proteins, such as E-cadherin and β-catenin, while mesenchymal proteins such as vimentin and N-cadherin are up-regulated [6], [7]. After activation of the EMT -process, tumor cells lose their epithelial features, including cell adhesion and polarity, reorganize their cytoskeleton, and acquire a mesenchymal morphology and the ability to migrate [5]. Furthermore, researchers have also shown that EMT is associated with the dedifferentiation program that leads to malignant carcinoma [8]. The identification of EMT enhancers and the underlying signaling pathways may improve our understanding of the EMT process and provide future targeted therapy for OSCC patients.

Neuropilin-1 (NRP1) is a transmembrane glycoprotein expressed by endothelial, dendritic, and regulatory T cells, as well as several other normal cell types and malignant tumor cells [9]–[16]. NRP1 was first identified as a semaphorin (SEMA) receptor, involved in axonal guidance in embryonic development [17]–[19]. Subsequently, NRP1 was also shown to act as a receptor for multiple members of the vascular endothelial growth factor (VEGF) family and a promoter of angiogenesis through its interaction with VEGF-A165 (and other VEGFs) and the receptor tyrosine kinase (RTK) VEGF-R2 [20]–[22]. In addition to its function in neural and vascular development, NRP1 expression has been detected in various human cancers such as prostate carcinoma, pancreatic carcinoma, astrocytic tumors, and OSCC [14], [23]–[26]. In our previous studies [26], [27], we found that NRP-1 is significantly overexpressed in tongue squamous cell carcinoma (TSCC) tissues, compared with normal non-cancerous controls. We hypothesized a role for NRP1 as a differentiation-associated factor in the tumorigenesis of TSCC. Though NRP1 has been regarded as a valuable marker of human malignancies, the exact mechanisms invoked by NRP1 in tumorigenesis are less well defined.

Interestingly, NRPs were recently reported to be involved in EMT in several studies [28], [29], with the evidence indicating the presence of elevated levels of the specific epithelial markers in renal cell carcinoma (RCC) cells in which NRP-1 expression was knocked down, with a concomitant decrease in the expression of specific mesenchymal markers [30]. Similar to these observations, in our recent study in SCC25 cells, NRP-1 down-regulation through sh-RNA-mediated methods promoted elevation of epithelial markers [27]. However, the pathways through which NRP1 is involved in the regulation of EMT are mostly unknown.

Nuclear factor-kappa B (NF-κB) plays an essential role both in the induction and the maintenance of EMT and tumor metastasis [31]. In addition, NF-κB activation is also responsible for tumor mammosphere formation, which was shown to be dependent on NRP1 expression [32]. Taken together, these data support our hypothesis that NRP1 regulates the EMT process in OSCC through NF-κB activation.

In this study, we investigated the role of NRP1 as an enhancer of the EMT process, through NF-κB activation, in OSCC cells. We also examined the clinical significance of NRP1 expression in OSCC patients by evaluating the correlation between NRP1 expression and clinicopathological features of OSCC. The results indicate that NRP1 has a key role in tumor progression and that NRP1 expression is associated with lymph node metastasis.

## Materials and Methods

### Cell culture, plasmids, and transfection

The human OSCC cell line CAL27 was obtained from the American Type Culture Collection (ATCC). Human OSCC cell lines HN4 and HN6 [33] were generously donated by the Shanghai Ninth People's Hospital (Shanghai, China). Cells were cultured in a 1∶1 mixture of Dulbecco's Modified Eagle's medium and Ham's F12 medium containing 10% fetal bovine serum (FBS) supplemented with penicillin (100 U/mL) and streptomycin (100 mg/mL) at 37°C in an atmosphere of 5% CO_2_. The NRP1 expression vector (pcDNA3.1-NRP1), containing full-length NRP1 cDNA inserted into pcDNA 3.1, was a gift from Dr. Michael Klagsbrun and Prof. Patrice K. Donahoe (Department of Surgery [Pathology], Harvard Medical School Vascular Biology Program, Children's Hospital, Boston, MA, USA). For transfection, CAL27 cells (5×10^5^ cells per well in 6-well plates) were cultured to 80% confluence in complete growth medium, after which the medium was replaced with serum-free medium for 12–16 h. The purified pcDNA3.1 and pcDNA3.1-NRP1 plasmids were transfected into cells using Lipofectamine 2000 (Invitrogen) following the manufacturer's instructions. CAL27, HN4, and HN6 cells stably transfected with an empty vector (referred to as CAL27-P, HN4-P, and HN6-P, respectively) and CAL27-pcDNA3.1-NRP1, HN4-pcDNA3.1-NRP1, HN4-pcDNA3.1-NRP1 cells (CAL27, HN4, and HN6 cells transfected with a vector encoding NRP1 [referred to hereafter as CAL27-N, HN4-N, and HN6-N cells, respeively]) were selected for 2 weeks with G418 (400 µg/mL; Invitrogen), which was added to the medium 48 h after transfection. Single Clones were selected and expanded, and successful transfection and stable expression of NRP1 was confirmed by western blotting analysis using anti-NRP-1 antibodies (see below). For experiments involving the addition of a specific NF-κB inhibitor, CAL27-N cells were treated with 5 µM pyrrolidinedithiocarbamate (PDTC; Sigma) and incubated for 24 h before carrying out further analyses.

### Patients and sample collection

A total of 60 primary OSCC cases were histopathologically and clinically diagnosed in the Stomatologic Hospital of Jiangsu Province, Nanjing Medical University between 2000 and 2008. None of the patients had been treated with any tumor-specific therapy before surgery. The follow-up period ranged from 4 to 116 months after surgical resection (average: 49.8 months; median: 51.0 months). Patients who died of other causes were excluded from the analysis. Tissue samples from non-cancerous oral lesions were also collected during the study period and served as the normal controls. Among the 30 normal tissue samples, 13 were obtained from the defect border after removal of the benign oral neoplasms and 17 were obtained from freshly injured oral mucosa after trauma. All tissues were obtained with the consent of the patients and written informed consent from the donors or the next of kin was obtained for use of the tissue samples for research purposes. This study was approved by the institutional ethics committee of the Nanjing Medical University.

### Real-time reverse transcription (RT)-PCR

Total RNA were extracted from cells using Trizol reagent (Invitrogen). Reverse transcription reactions were performed using the 5× PrimeScript RT Master Mix (TaKaRa) at 37°C for 15 min and 85°C for 5 s, according to the manufacturer's protocol. Quantitative PCR was carried out with 2× SYBR Premix Ex Taq (TaKaRa) in the ABI-7300 Real-Time PCR System (Applied Biosystems) for 40 cycles under the following conditions: 95°C 30 s, then 95°C5 s, and 60°C 31 s. The relative gene expression was analyzed by the 2(−ΔΔCT) method, with glyceraldehyde 3-phosphate dehydrogenase (GAPDH) as an internal control. The sequences for sense and antisense primers are as follows:

NRP1: 5′-CAGGTGATGACTTCCAGCTCA-3′ (sense) and 5′-CCCAGTGGCAGAAGGTCTTG-3′ (antisense);

E-cadherin: 5′-AAG AAA ACCCGAAGAGG-3′ (sense) and 5′-CTGACTCAAGGTGCA GC-3′ (antisense);

β-catenin: 5″-CCCACTAATGTCCAGCGTTT-3′ (sense) and 5′-AATCCACTGGTGAACCAAGC-3″(antisense);

N-cadherin: 5′-GACGGTTCGCCATCCAGAC-3′ (sense) and 5′-TCGATTGGTTTGACCACGG-3′ (antisense);

vimentin: 5′-TGA GTA CCG GAG ACA GGT GCA G-3′ (sense) and 5′-TAG CAG CTT CAA CGG CAA AGT TC-3′ (antisense);

GAPDH: 5′-GAA GGT GAA GGT CGG AGT C-3′ (sense) and 5′-GAG ATG GTG ATG GGA TTT C-3′ (antisense).

### Western blotting

Total protein was extracted from cells using lysis buffer (Beyotime), and the cytoplasmic and nuclear extracts were obtained using the Nuclear and Cytoplasmic Extraction Reagents (Keygen Biotech). Coomassie Brilliant Blue was used to quantify the protein content, using bovine serum albumin as the standard. The proteins (10 µg) were resolved using sodium dodecyl sulfate-polyacrylamide gel electrophoresis (SDS-PAGE) with 10% polyacrylamide gels and then transferred to polyvinylidene difluoride (PVDF) membranes (Millipore), which were blocked with 5% nonfat milk in phosphate-buffered saline (PBS) containing Tween-20 (PBS-T) for 2 h at room temperature. The blots were then probed with primary antibodies specific for NRP1 (1∶1000; Abcam), E-cadherin (1∶1000; Bioworld), β-catenin (1∶1000; Bioworld), N-cadherin (1∶1000; Abcam), vimentin (1∶1000; Bioworld) and β-actin (1∶1000; Bioworld) overnight at 4°C, washed twice with PBST, and incubated with horseradish peroxidase-conjugated (HRP) secondary antibodies for 1 h at room temperature. Finally, the protein bands were detected using an Immobilon Western Chemiluminescent HRP Substrate (Millipore) and visualized using the ImageQuantLAS 4000 mini imaging system (General Electrics). Three independent trials of each experiment were carried out. Densitometric analysis of the scanned blots was performed using Image J software. The results were expressed as fold-change relative to β-actin levels, which served as the internal control.

### Immunofluorescence assays

Cells cultured on glass coverslips were washed with PBS, fixed in 4% paraformaldehyde (PFA) for 30 min at room temperature. The cells were then permeabilized with 1% Triton X-100 for 15 min and blocked with 1% bovine serum albumin (BSA) for 30 min, bound with antibodies specific for β-catenin (1∶100, Proteintech Group) at 4°C overnight. FITC-labeled goat anti-rabbit IgG were used as the secondary antibody (dilution, 1∶50). Nuclei were then stained by 4, 6-diamidino-2-phenylindole (DAPI; 1∶1000, Invitrogen) for 1 min. Immunofluorescence was analyzed with fluorescence microscopy.

### Scratch assays

Cells were cultured in 6-well plates to 90% confluence and then scratched with a sterile 200 µL pipette tip in the central area. Floating cells and debris were carefully removed with PBS, and the culture medium was replaced with a serum-free medium. Migration of wounded cells was observed under a microscope and images of the same wound area were captured over time.

### Invasion Assays

The invasion ability of cells was analyzed using Transwell filters (8 µm pore size; Millipore) coated with 50 µL Matrigel Basement Membrane Matrix (BD Biosciences). The cells (5×10^4^) were plated in 200 µL serum-free medium, inoculated in the upper chamber; while 500 µL of medium containing 10% FBS was used as the chemoattractant was placed in the lower chamber. After incubating the cells for 24 h at 37°C, the non-invading cells remaining on the upper side of the filter were gently removed with cotton swabs. The invading cells on the lower membrane were fixed with 4% paraformaldehyde (PFA) for 10 min and stained with crystal violet for 30 min. The stained invaded cells in five random fields at ×200 magnification were manually counted, and the mean cell count was calculated to obtain the proportion of invasive cells.

### Colony-formation assay

Cells were seeded in 60-mm dishes (Corning) at a density of 1000 (CAL27 and HN6)/3000 (HN4) cells per dish and cultured at 37°C for 14 days. The cells were fixed in 4% PFA, stained with crystal violet, and images were captured. Aggregates of more than 50 cells were defined as a colony and the colonies were observed and counted under an inverted microscope (Olympus).

### Flow cytometry

For analysis of apoptosis, the cells were treated with 5 or 10 µg/mL cisplatin for 24 h. Then, the drug-treated cells were harvested. Flow cytometric analysis of apoptosis was performed by staining cells with Annexin V-fluorescein isothiocyanate (FITC) and propidium iodide (PI) using the Annexin V Apoptosis Detection Kit I (BD Pharmingen) for 15 min according to the manufacturer's protocol. The proportion of apoptotic cells was determined with a FACS Calibur flow cytometer (BD Biosciences) and the CellQuest Pro software (BD Biosciences). The rate of apoptosis in the drug-treated cells was compared with that of untreated cells, which served as the negative control.

### Immunohistochemistry

Immunohistochemical analysis was performed to study NRP1 expression in 60 paraffin-embedded tissues which had been processed into 5-µm serial sections as described previously [27]. Briefly, the sections were incubated with a rabbit monoclonal anti-NRP1 antibody (1∶100; Abcam) with the Two-Step Histostaining Reagent (ZhongshanGoldenbridge Bio). Negative control slides were prepared by omitting the primary antibody under the same experimental conditions, and the absence of non-specific immunoreactive staining was confirmed.

Immunoreactivity was semi-quantitatively evaluated on the basis of staining intensity and distribution scores [26]. Stromal NRP1 expression was not considered in the quantification of NRP1 staining in tumors. The intensity score was defined as 0, negative; 1, weak; 2, moderate; or 3, strong, and the proportion score was defined as 0, negative; 1, <10%; 2, 11–50%; 3, 51–80%; or 4,>80% positive cells. The immunoreactivity score, which defined as the product of the proportion score multiplied by the intensity score, ranged from 0 to 12. The sections were divided into the following three groups based on the immunoreactivity score: negative immunoreactivity was defined as a total score of 0, low expression was defined as a total score of 1–4, and high expression was defined as a total score >4.

### Statistical analyses

Statistical analyses were performed using the SPSS software (version 19.0). Results of quantitative data from at least three independent trials of each experiment were expressed as the mean ± SD and evaluated using the Student's t-test. Ratio analysis was performed with the χ^2^ or Fisher's exact tests. The survival index was obtained using the Kaplan-Meier method and compared using the log-rank test. A value of P<0.05 was considered to be statistically significant.

## Results

### NRP1 overexpression mediates morphological changes of OSCC cells and induces EMT

**Figure 1:**
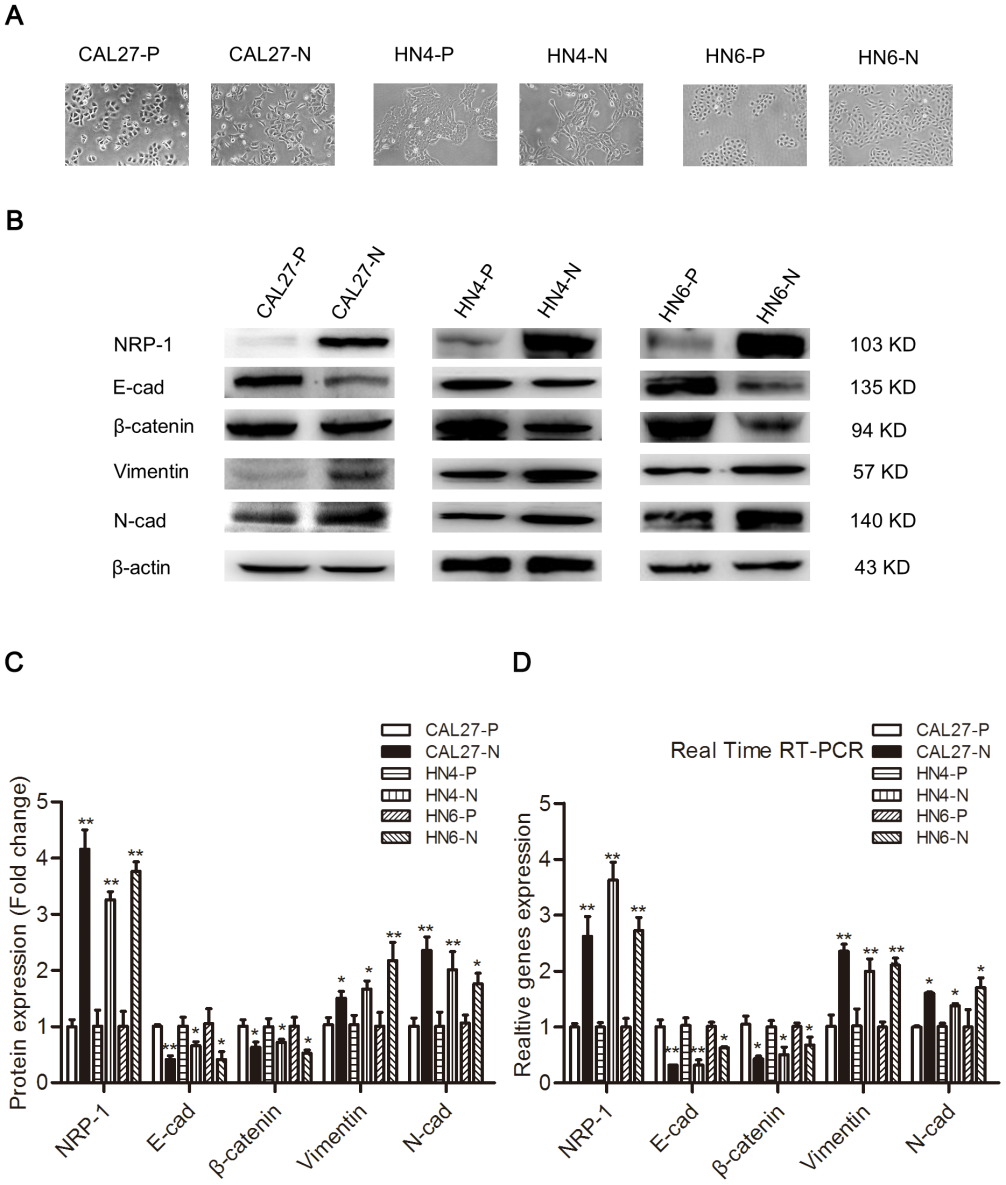
NRP1 induces the EMT process in OSCC cell lines. (**A**) CAL27, HN4, and HN6 cells transfected with the vector control (CAL27-P, HN4-P, and HN6-P) had a typical pebble-like epithelial morphology, while cells transfected with the NRP1-expressing plasmid (CAL27-N, HN4-N, and HN6-N) had a more elongated, pebble-like morphology. The images were captured using an inverted microscope (Olympus) fitted with a camera. The images were captured at ×40 magnification; Scale bar, 100 µm. (**B, C**) Western blotting and (**D**) Real time RT-PCR analyses were used to assess the expression of epithelial (E-cadherin and β-catenin) and mesenchymal (N-cadherin and vimentin) markers in OSCC cells transfected with the vector control or a vector encoding NRP1. β-actin and GAPDH were employed as controls in the western blotting and RT-PCR analyses, respectively. Each data point represents the mean ± SD of three independent repetitions of the experiment. *P<0.05; **P<0.01.

To examine whether NRP1 promotes EMT in OSCC cells, we stably transfected control and human NRP1-encoding vectors into CAL27, HN4, and HN6 cells. OSCC cells transfected with an empty vector (CAL27-P, HN4-P, and HN6-P) exhibited a typical pebble-like epithelial morphology and cell-cell adhesions were detected clearly (Figure 1A). However, cells transfected with a vector encoding NRP1 (CAL27-N, HN4-N, and HN6-N) had a more spindle-like shape, along with a decrease in cell-to-cell contact (Figure 1A). Western blotting and real-time RT-PCR analyses were utilized to assess changes in the protein and mRNA levels of EMT-specific markers. As anticipated, NRP1 overexpression caused the down-regulation of the epithelial markers E-cadherin and β-catenin, while the expression of mesenchymal markers N-cadherin and vimentin were increased (Figure 1B–D). Down-regulation of the epithelial marker β-catenin on the cytomembrane were further detected by Immunofluorescence assays (Figure S1A–C). Our data indicated that NRP1 overexpression induced expression changes consistent with EMT in OSCC cells.

### Overexpression of NRP1 alters the biological behavior of OSCC cells

**Figure 2:**
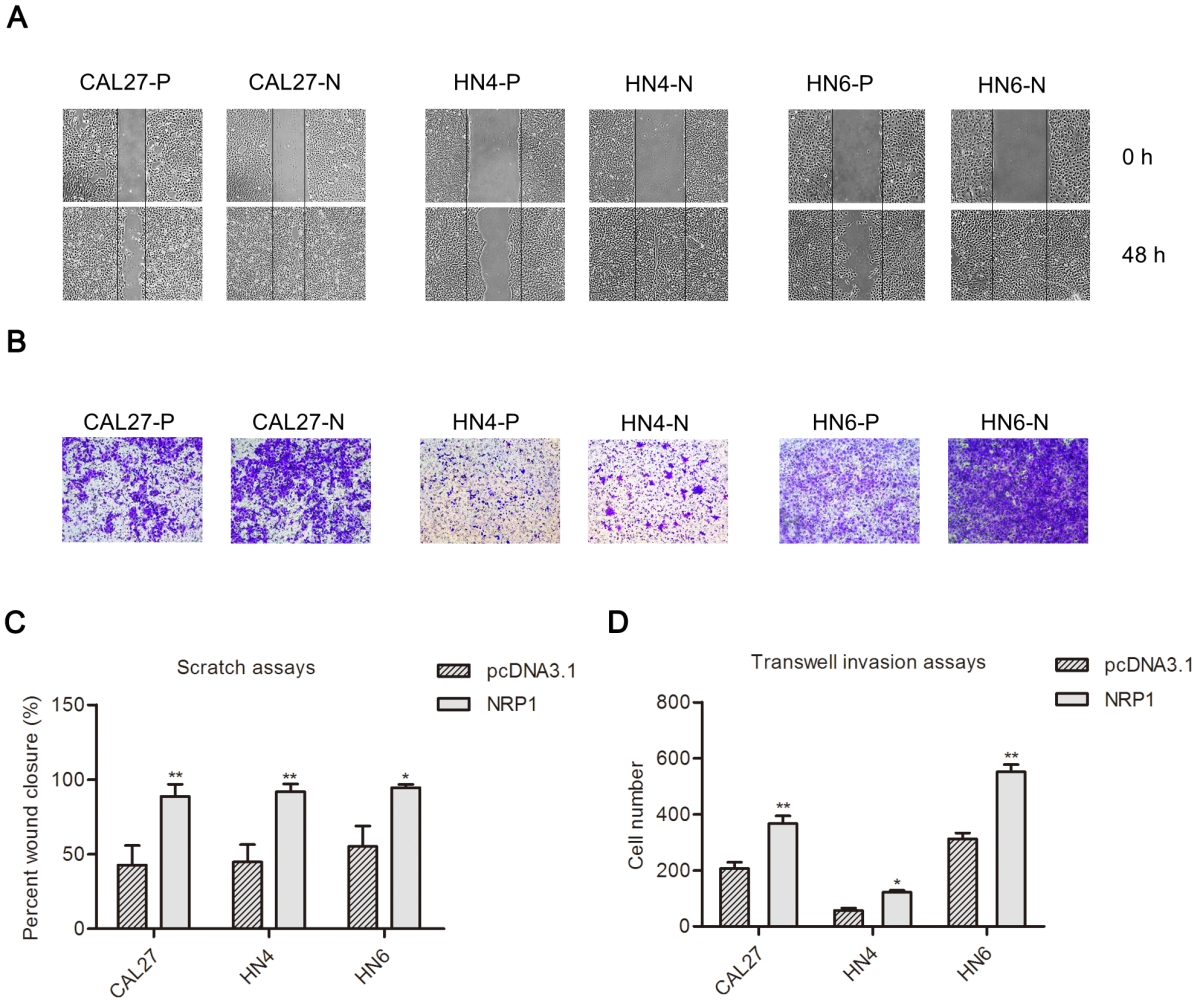
The examination of the biological properties of OSCC cells transfected with an empty vector or a vector encoding NRP1. (**A**) The differences in the migration ability between the CAL27-P, HN4-P, and HN6-P and CAL27-N, HN4-N, and HN6-N cells, respectively, were measured using the scratch assay. The CAL27-N, HN4-N, and HN6-N cells grew into the wounded area after 48 h, while CAL27-P, HN4-P, and HN6-P cells did not. The images were captured at ×40 magnification; Scale bar, 100 µm. (**B**) A transwell assay was employed to analyze the cell invasion ability. CAL27-N, HN4-N, and HN6-N cells had significantly higher invasion ability compared with CAL27-P, HN4-P, and HN6-P cells. The images were captured at ×200 magnification; Scale bar, 100 µm. Quantitative analysis of the data from the (**C**) scratch assay after 48 h and (**D**) transwell invasion assays after 24 h in five randomly-selected fields. The data shown are the mean ± SD. *P<0.05; **P<0.01.

Next, we investigated whether NRP1-induced EMT in mesenchymal cells promoted their migratory and invasive abilities using scratch and transwell assays. As shown in Figure 2A, the cell-free area of the CAL27-N, HN4-N, and HN6-N cells filled the scratch at 48 h, while the scratched region persisted in CAL27-P, HN4-P, and HN6-P cells (Figure 2A and C). Consistent with these observations, overexpression of NRP1 significantly induced cell invasion in the reconstituted basement membrane (Matrigel), compared with the control CAL27-P, HN4-P, and HN6-P cells (Figure 2B and D). These results demonstrate that NRP1 plays a functional role in mediating cell migration and invasion in OSCC cells.

### Effects of NRP1 overexpression on cell proliferation and resistance to therapeutic drugs

**Figure 3:**
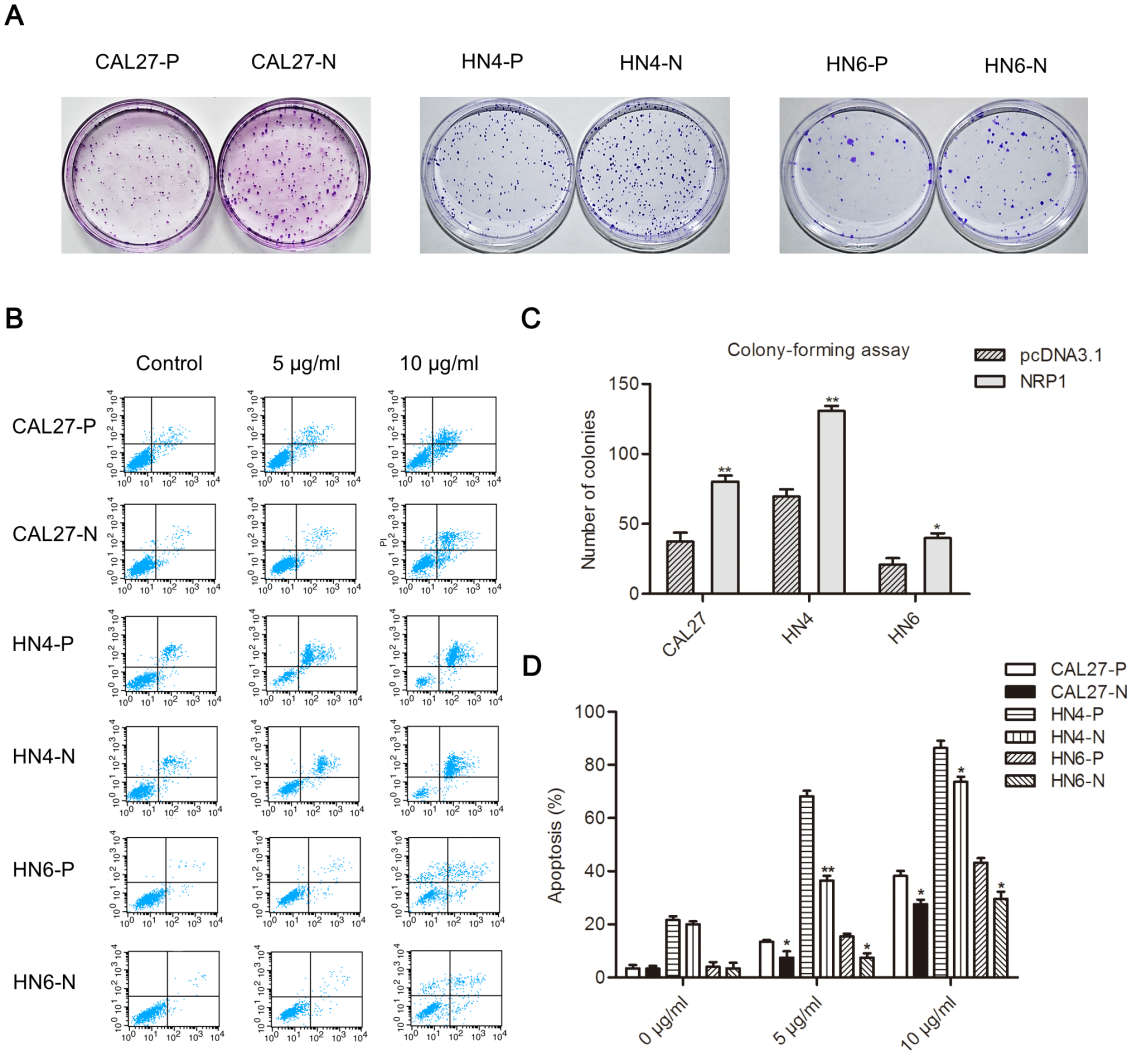
NRP1 may play a role in the regulation of proliferation and drug resistance in OSCC cells. (**A**) Images of colonies of CAL27-P/N, HN4-P/N, and HN6-P/N cells stained with crystal violet. (**B**) Flow cytometric analysis of apoptosis in CAL27-P/N, HN4-P/N, and HN6-P/N cells treated with different concentrations of cisplatin for 24 h. (**C**) Quantification of cell colonies. Each data point represents the mean ± SD of data from three independent trials. *P<0.05; **P<0.01. (**D**) Statistical analysis of the rate of cell apoptosis in CAL27-P/N, HN4-P/N, and HN6-P/N cells. Each bar represents the mean ± SD of data from three independent trials. *P<0.05; **P<0.01.

Next, colony-formation assays were performed to determine the effect of NRP1 on cell proliferation. The data showed that CAL27-N, HN4-N, and HN6-N cells exhibited a higher colony- formation capability compared with CAL27-P, HN4-P, and HN6-P cells (Figure 3A and C), suggesting that NRP1 promotes OSCC cell proliferation. Furthermore, the effect of NRP1 overexpression on drug resistance in OSCC cells was assessed by treating cells with cisplatin, a chemotherapy drug. After treatment of the cells with different concentrations of cisplatin (5 and 10 µg/mL) for 24 h, cell apoptosis was analyzed using flow cytometry. As shown in Figure 3B and D, CAL27-N, HN4-N, and HN6-N cells had a lower rate of apoptosis than CAL27-P, HN4-P, and HN6-P cells, which indicated that NRP1 overexpression may contribute to the elevated drug resistance of OSCC cells.

### NRP1-mediated EMT occurs through activation of the NF-κB signaling pathway

**Figure 4:**
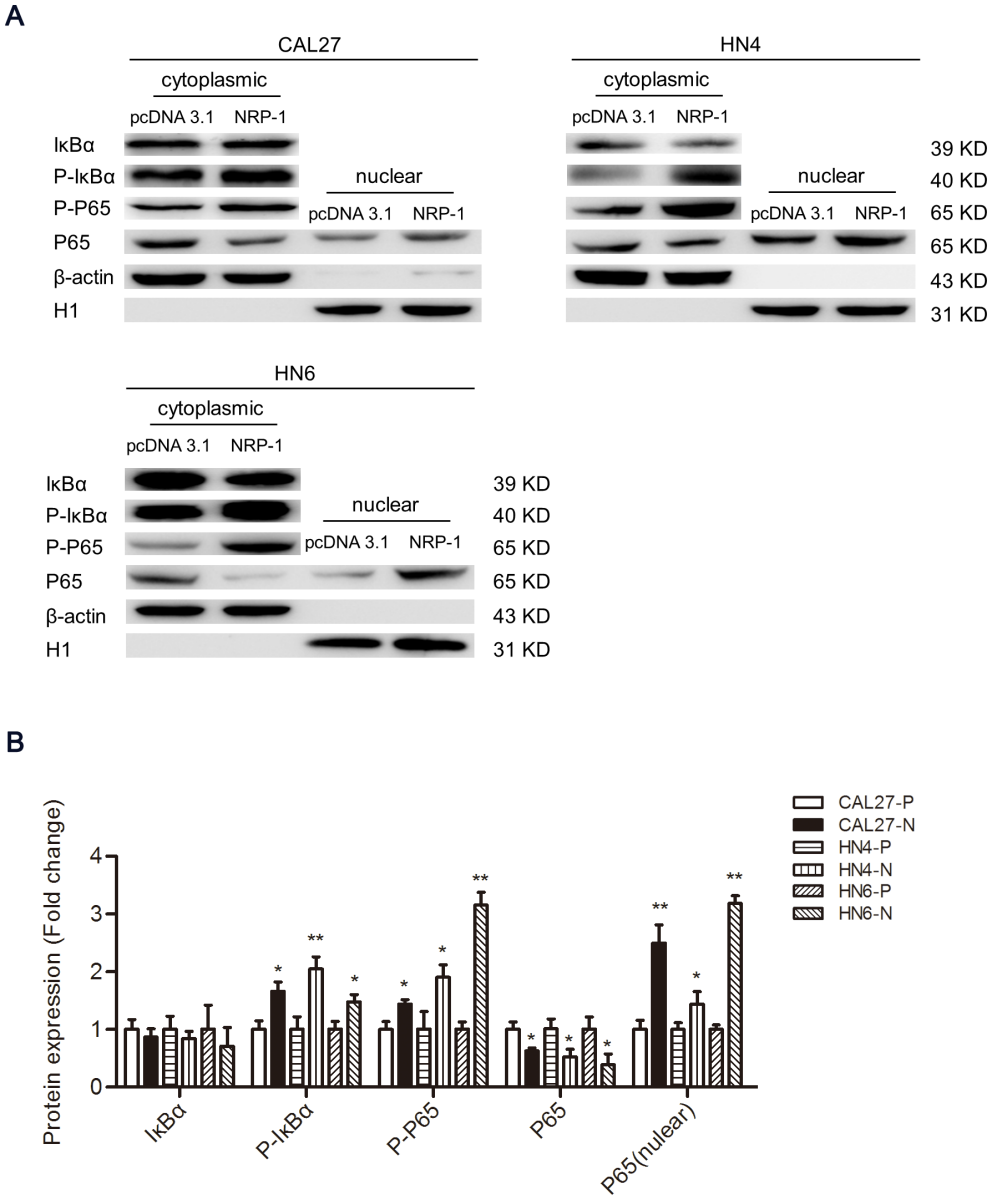
NRP1-mediated EMT occurs through activation of the NF-κB signaling pathway. (**A**) Western blotting was performed to assess the expression level of NF-κB pathway-related proteins in cytoplasmic and nuclear extracts from CAL27-P/N, HN4-P/N, and HN6-P/N cells. β-actin and histone H1 were employed as the positive controls for cytoplasmic and nuclear proteins, respectively. (**B**) Semi-quantitative analysis of changes in protein expression as determined by densitometric scanning of the immunoreactive bands. Each data point represents the mean ± SD of three independent trials. *P<0.05; **P<0.01.

Previous studies have indicated that the induction of EMT may be an important mechanism of constitutive NF-κB signaling activation in various cancers [32], [34]. To further explore whether the NRP1-mediated EMT process in OSCC cell lines is dependent on the activation of the NF-κB pathway, western blotting analysis was conducted to assess the activation of the components of the NF-κB pathway in NRP1- overexpressing OSCC cells. Nuclear translocation of the p65 subunit of NF-κB and phosphorylation of inhibitor of NF- κB (IκB)α was detected in NRP1-overexressing OSCC cells, demonstrating that NRP1 overexpression results in a marked increase of NF-κB signaling activation in CAL27, HN4, and HN6 cells (Figure 4 A and B).

**Figure 5:**
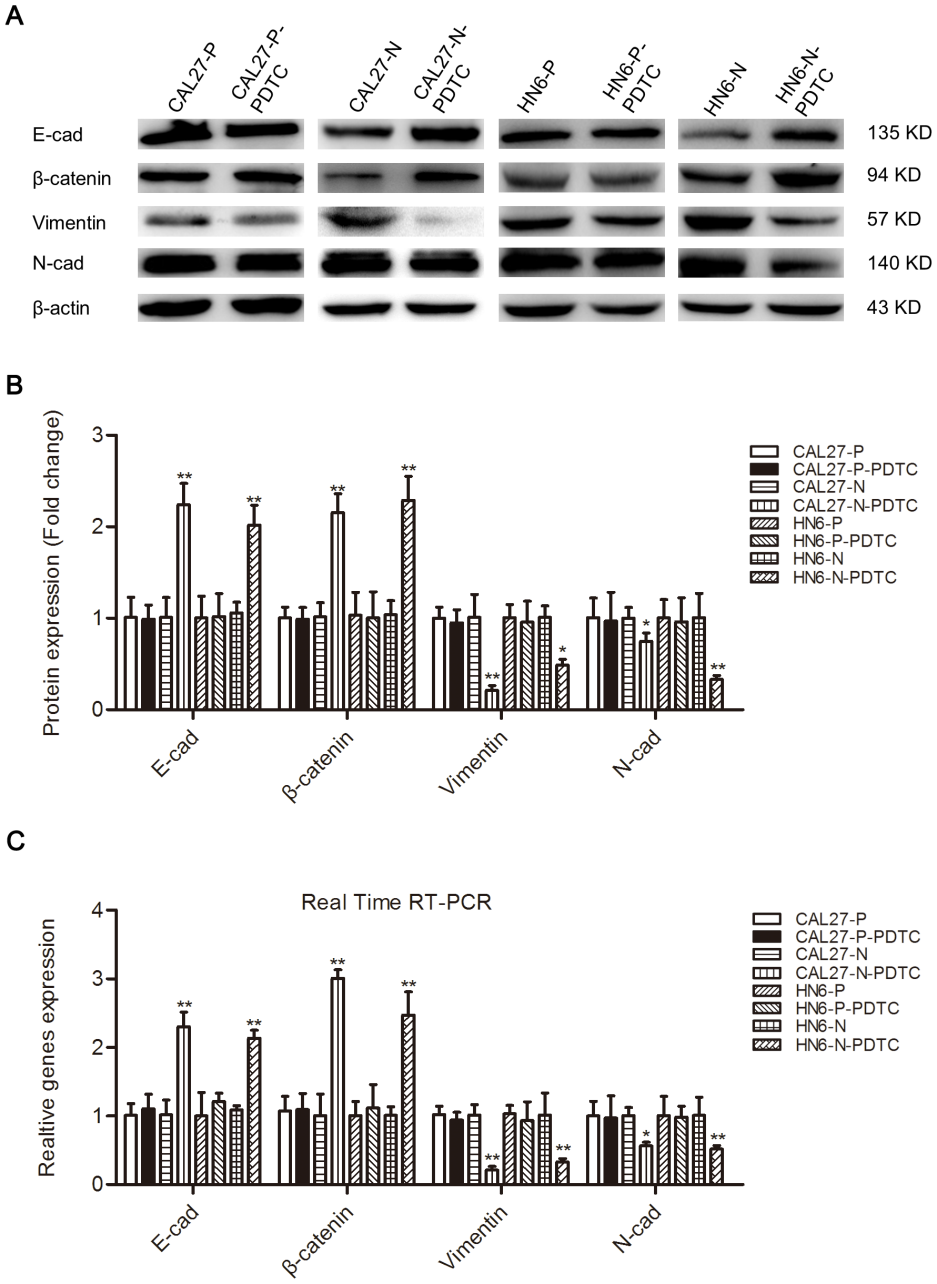
Inhibition of the NF-κB signaling pathway results in a reversion of NRP1-mediated EMT. After treatment with PDTC for 24(**A, B**) western blotting and (**C**) Real time RT-PCR was used to detect the expression of EMT-related proteins in CAL27-P/N, HN4-P/N, and HN6-P/N cells. β-actin and histone H1 were employed as controls. Each data point represents the mean ± SD of three independent trials. *P<0.05; **P<0.01.

**Figure 6:**
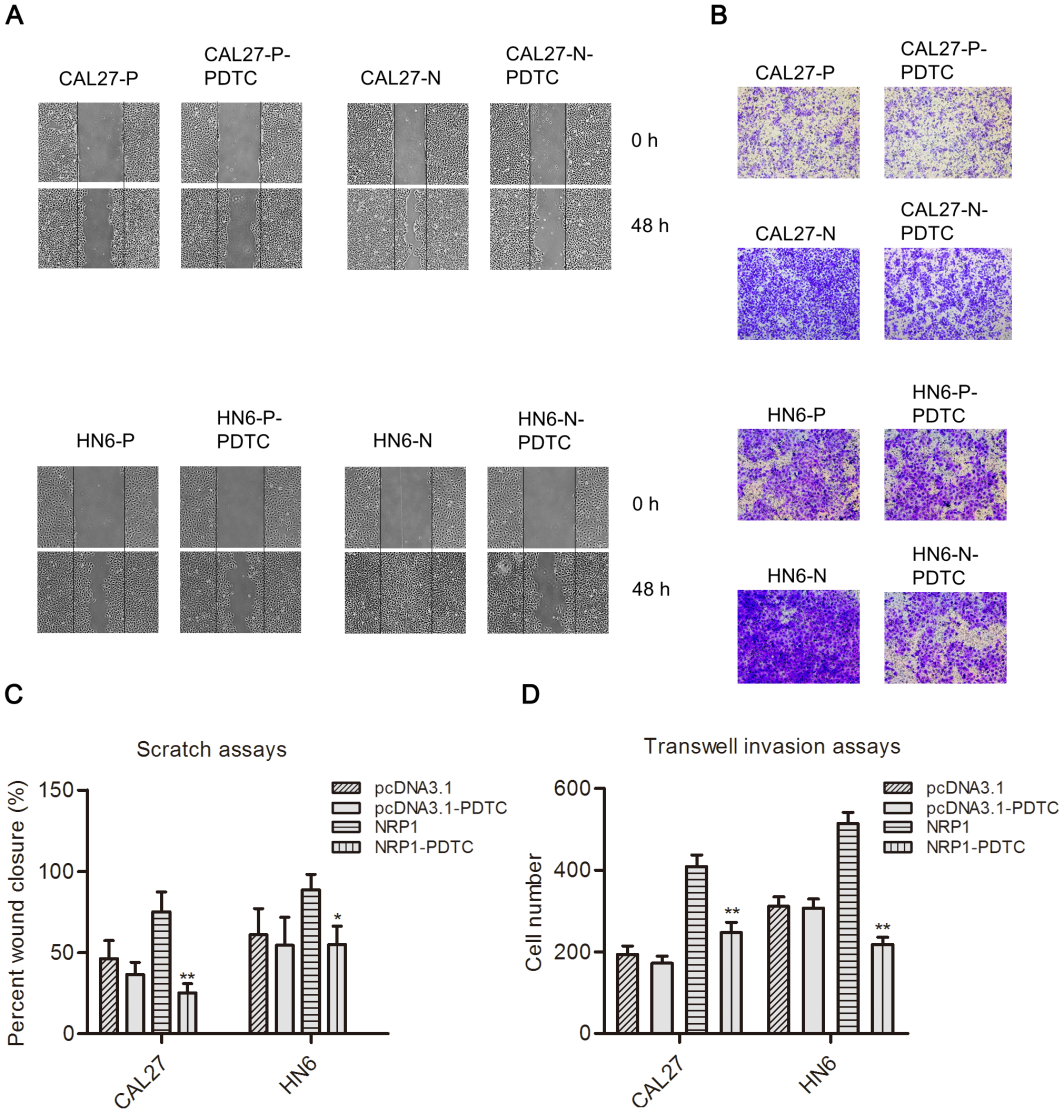
Inhibition of NF-κB signaling pathway alters the biological properties of NRP1-expressing OSCC cells. (**A**) Scratch assays were performed to examine the migration ability in CAL27-P/N, HN4-P/N, and HN6-P/N cells, treated with vehicle control or PDTC for 24 h. Images of the wound area were captured at ×40 magnification; Scale bar, 100 µm. (**B**) Transwell assays with a reconstituted basement membrane were performed to examine the invasion ability in CAL27-P/N, HN4-P/N, and HN6-P/N cells, treated with vehicle control or PDTC for 24 h. The images were captured at ×200 magnification; Scale bar, 100 µm. Quantitative analysis of data from the (**C**) scratch assays after 48 h and (**D**) cell invasion assays after 24 h in five randomly-selected fields. The data shown are the mean ± SD of three independent trials. *P<0.05; **P<0.01.

We also addressed the role of the NF-κB pathway in NRP1-medated EMT using a specific NF-κB inhibitor, PDTC. CAL27 and HN6 cells were treated with PDTC, and the effects on the expression of EMT markers were evaluated using western blotting analysis and real-time RT-PCR. As expected, after treatment with PDTC, the expression of E-cadherin and β-catenin was increased, while N-cadherin and vimentin expression was down-regulated considerably in OSCC cells overexpressing NRP1, while no significant change were observed in CAL27-P or HN6-P cells with or without PDTC treatment (Figure 5 A–C). These data suggest that NF-κB inhibition results in a reversion of the NRP1- mediated EMT process. Scratch and transwell invasion assays were also utilized to assess the changes in the biological behaviors of OSCC cells after treatment with PDTC. The PDTC-treated NRP1-overexpressing cells displayed significantly lower migration and invasion abilities than the corresponding parental cell lines, while no significant changes were detected in the migration or invasiveness CAL27-P or HN6-P cells with or without PDTC treatment (Figure 6 A–D). These data are consistent with the hypothesis that NRP1-mediated EMT occurs through and/or is dependent on activation of the NF-κB signaling pathway.

### Higher expression of NRP1 is observed in OSCC patient-derived tissues and correlates with lymph node metastasis and poor prognosis

**Figure 7:**
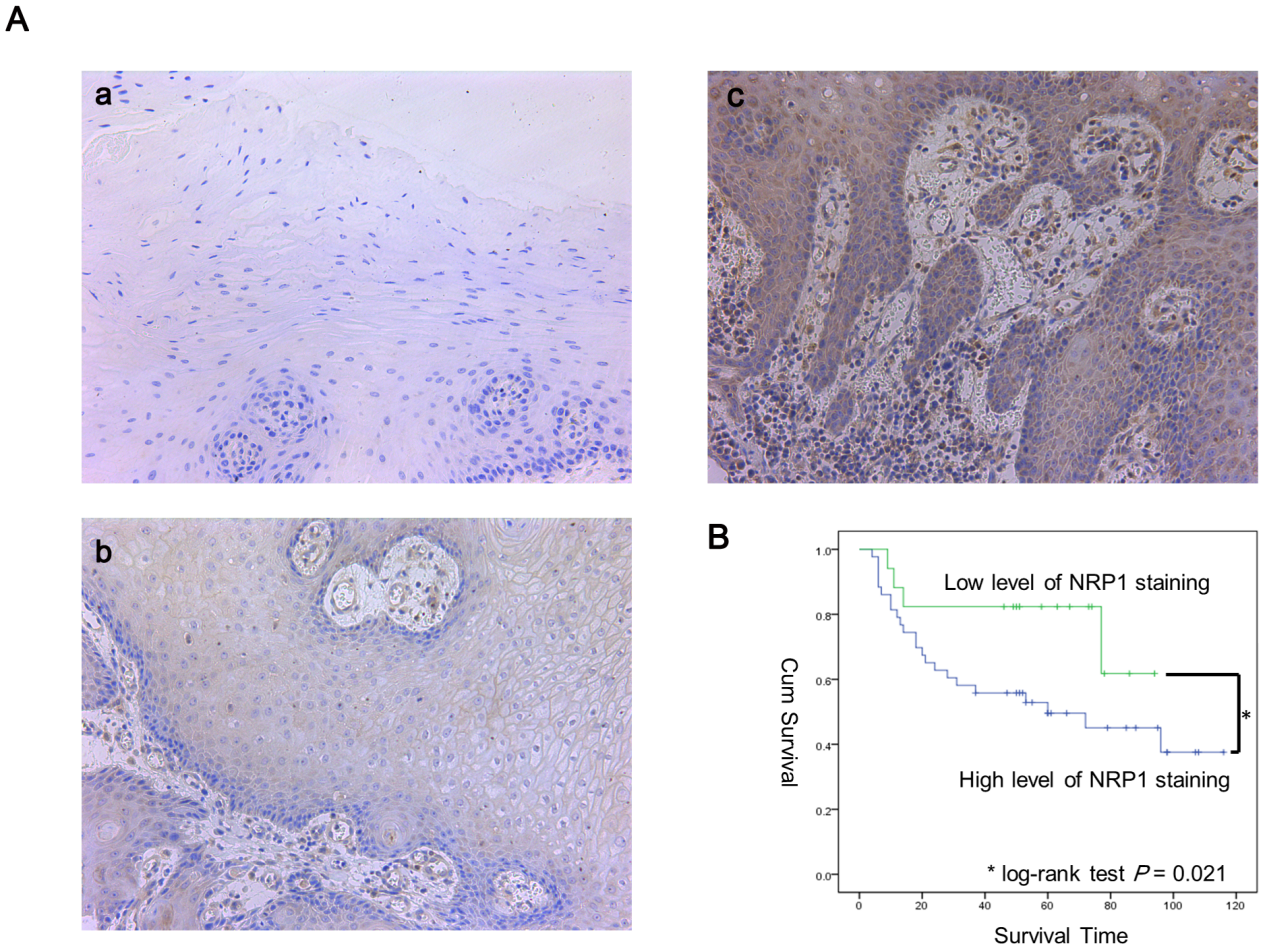
High expression of NRP1 correlates to lymph node metastasis and poor prognosis in OSCC patients. (**A**) The expression of NRP1 in normal oral epithelium (a), a tissue sample from a patient without lymph node metastasis (b), and a tissue sample from a patient with lymph node metastasis (c) (magnification, ×200). (**B**) Kaplan-Meier overall survival curves for 60 patients with OSCC, according to NRP1 expression. *P<0.05.

To examine the correlation between the expression level of NRP1 in OSCC tissues and its influence on cancer metastasis and disease prognosis, the expression of NRP1 in 90 paraffin-embedded tissue samples, consisting of 60 primary OSCC tissues and 30 non-cancerous controls, were assessed by immunohistochemical analysis. Among the 90 samples, positive staining for NRP1 was observed in all of the tumor specimens, while only 6 cases were positive for NRP1 expression among the 30 normal oral epithelial tissues evaluated (Table 1). Furthermore, among the tumor specimens, there was a significant correlation between higher NRP-1 expression levels and lymph node metastasis (P = 0.007; Table 2 and Figure 7A). There was no correlation between high levels of NRP1 staining and age, sex, or tumor T stages (Table 2). We then analyzed the prognostic data of these patients. Consistent with the data pertaining to lymph node metastasis, poorer prognosis was observed in patients with OSCCs that had high NRP1 expression, compared to those with tumors expressing lower levels of NRP1 (Figure 7B).

**Table 1:** Expression of NRP1 in normal oral epithelium and OSCC.

	No.	NRP1			P-value
		N	L	H	
tumor	60	0	17	43	**<0.001** *
normal	30	24	5	1	

N, negative; L, low expression; H, high expression.

*P<0.05.

**Table 2:** Association between OSCC clinical-pathological parameters and NRP1 expression.

		NRP1		
Clinicopathologic Characteristic	No.	L	H	P-value
Age				0.386
≤50	14	3	11	
>50	46	14	32	
Sex				0.5
Male	37	11	26	
Female	23	6	17	
tumor stage				0.565
I–II	31	9	22	
III–IV	29	8	21	
T Stage				0.255
T1–T2	33	11	22	
T3–T4	27	6	21	
N Stage				**0.007** *
N0	33	14	19	
N+	27	3	24	

N, negative; L, low expression; H, high expression; N0, no nodal metastasis; N+, nodal metastasis.

*P<0.05.

## Discussion

NRP1 is a multifunctional type I transmembrane glycoprotein that plays an important role in axonal extension, angiogenesis, and cancer progression [17]–[22], [29]. Numerous reports indicate that NRP1 may serve as a potential prognostic biomarker and a valuable therapeutic target for a variety of human cancers [32], [35], [36]. In this study, we investigated the roles played and functional mechanisms invoked by NRP1 in OSCC progression. Our previous study showed that the expression of NRP1 was up-regulated in human TSCC tissues and that NRP1 overexpression may be responsible for the poor prognosis of individuals with a high NRP1/SEMA3A expression ratio [26]. Interestingly, we also found that after NRP1 knockdown in SCC25 cells, the mesenchymal marker vimentin was significantly down-regulated, while E-cadherin, an epithelial marker, was up-regulated, indicating that NRP1 could be an enhancer of EMT in OSCC [27]. Thus, in the present study, we focused on the connection between NRP1 expression and EMT. Our data show that up-regulation of NRP1 can promote EMT in OSCC cells, and that the NRP1-mediated changes in cell morphology viand function are dependent on NF-κB activation.

Upon overexpression of NRP1, CAL27-N, HN4-N, and HN6-N cells displayed a more spindle-like morphology, compared with the cubic epithelial phenotype of CAL27-P cells. The induction of EMT in CAL27-N, HN4-N, and HN6-N cells was confirmed by the loss of epithelial markers (E-Cadherin and β-catenin) and an increase of mesenchymal markers (vimentin and N-Cadherin) at both the mRNA and protein levels. Previous studies showed that NRP1 overexpression in several malignances may contribute to local tumor invasiveness and migration [35], [36]. We investigated the changes in the biological behavior of the OSCC cells overexpressing NRP1. Our results confirm that NRP1 promotes cancer cell migration and invasion in OSCC through induction of the EMT process.

Sustained proliferation and resistance to apoptosis are considered to be classical CSC hallmarks crucially involved in the pathogenesis of malignant neoplasias [37], [38]. A study by Santisteban et al. [39] suggests that the in vitro induction of EMT can cause the acquisition of CSC properties in differentiated epithelial tumor cells. Importantly, tumor stem cells are believed to escape from anti-cancer drug targeting and are resistant to apoptosis [40]. In the current study, our data show that NRP1 could have a role in the regulation of proliferation and drug resistance in OSCC, implicating NRP1 as a potential promoter for some CSC-like properties in mesenchymal cells, which is in accordance with the conclusions of Glinka et al. [32] in breast cancer stem-like cells and of Beck et al. [41] in cutaneous CSCs.

NF-κB was originally identified as a transcriptional regulator of inflammatory and innate immune responses. Further studies revealed that NF-κB plays an important role in oncogenesis, cell proliferation, and cell migration [42]. NF-κB has been hypothesized to play a key role in maintenance of EMT, principally via the phosphorylation of IκBα and nuclear translocation of the p50/p65 heterodimer, which then alter the expression of genes that maily regulate cell survival, proliferation, and inflammation [31], [32]. Recent studies have revealed that NF-κB could induce EMT in several malignancies, including non-small cell lung and breast cancers [34], [43]. To further elucidate the molecular mechanisms of NRP1-induced EMT in OSCC, we evlauated NF-κB activation in NRP1-overexpressing OSCC cells. The data showed that IκBα is phosphorylated and p65 is translocated into the nucleus in NRP1-overexpressing OSCC cells. Treatment with PDTC, a specific NF-κB inhibitor, reversed NRP1-induced EMT and abrogated the NRP1-mediated increase in the metastatic and invasive capability of OSCC cells. Based on these findings, we conclude that the NF-κB signalling pathway is involved in the NRP1-mediated induction of EMT, migration, and invasion in OSCC cells.

We extended these in vitor studies to human OSCC by evaluating the correlation between NRP1 expression and clinicopatholgical features and prognosis of OSCC in human patients. We found that NRP-1 was highly expressed in tumor specimens, compared with normal (non-cancerous) oral tissues. Moreover, high expression of NRP1 was significantly correlated to lymph node metastasis and poor prognosis in OSCC patients. These finding are partially consistent with those of previous reports in non-small cell lung carcinoma and colorectal cancer [35], [44]. A study by Ochiumi et al. [34] indicated that NRP1 induced tumor growth and migration in colon carcinoma through interaction with its ligands, VEGF165 and SEMA [35]. Our previous study also showed that the ratio of NRP1 to SEMA3A was negatively correlated with survival in TSCC patients [26]. In our current study, we revealed a new mechanism, NRP1-mediated EMT, to explain the correlation between NRP1 overexpression and lymph node metastasis as well as poor prognosis in OSCC patients. In view of these data, analysis of NRP1 expression in surgical tissue samples from OSCC patients may provide additional information pertinent to the decision-making process regarding appropriate treatment strategies in OSCC. Given the correlation between high NRP1 expression and poor prognosis, patients with tumors expressing high levels of NRP1 may need to be treated with more aggressive therapies or may benefit from therpaies targeting the NF-κB pathway. Further in vivo studies assessing the correlation of NRP1 expression with prognois and metastasis in a larger cohort of OSCC patients are necessary to confirm the utlity of NRP1 as a viable target of OSCC therapies.

## Supporting Information

Figure S1 **Immunofluorescence analysis of epithelial marker β-catenin in OSCC cells.** Immunofluorescence staining of β-catenin (red) in (**A**) CAL27, (**B**) HN4, (**C**) HN6 cells. The nuclei were stained with DAPI. Images were taken at ×400 magnification. Bar, 100 µm
(TIF)
