# Supplementary figures and images for: Neuropilin-1 Promotes Epithelial-to-Mesenchymal Transition by Stimulating Nuclear Factor-Kappa B and Is Associated with Poor Prognosis in Human Oral Squamous Cell Carcinoma
Source: PLoS One. 2014 Jul 7;9(7):e101931. doi: 10.1371/journal.pone.0101931 (PMC4084996; doi:10.1371/journal.pone.0101931)

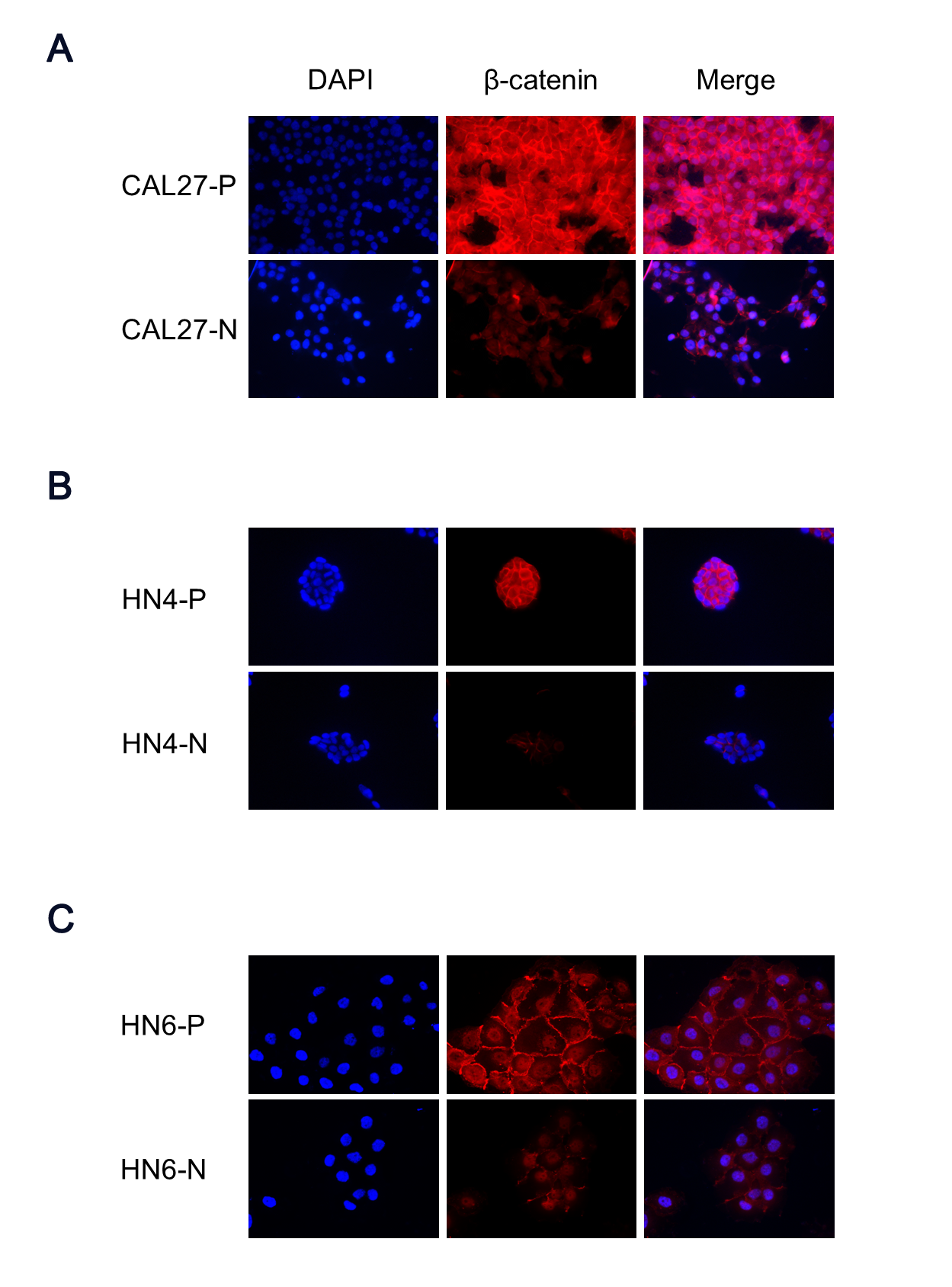

Supplement: Figure S1 — Immunofluorescence analysis of epithelial marker β-catenin in OSCC cells. Immunofluorescence staining of β-catenin (red) in (A) CAL27, (B) HN4, (C) HN6 cells. The nuclei were stained with DAPI. Images were taken at ×400 magnification. Bar, 100 µm (TIF) [file pone.0101931.s001.tif]
